# Done deal—cohabiting dominant and subordinate dogs differently rely on familiar demonstrators in a detour task

**DOI:** 10.1186/s12915-025-02232-9

**Published:** 2025-05-09

**Authors:** Péter Pongrácz, Petra Dobos, Fruzsina Prónik, Kata Vékony

**Affiliations:** Department of Ethology, ELTE, Eötvös Loránd University, Budapest, Hungary

**Keywords:** Hierarchy, Dog, Owner, Social learning, Dominant, Subordinate, Detour

## Abstract

**Background:**

Companion dogs live in a mixed-species environment, where they can successfully learn from both humans and dogs. Breed type, the demonstrator’s behavior, and in multi-dog households, the dogs’ hierarchy are known influencing factors of the efficiency of dogs’ social learning. In previous studies, always an unfamiliar dog or experimenter was the demonstrator of the given task. Now we tested social learning in a setting more relevant to the everyday life of dogs, where the demonstrator was either the owner or a cohabiting dog. We used the validated dog-rank assessment questionnaire (DRA-Q) and the well-established detour paradigm. We hypothesized that beyond the previously found associations between social learning and rank, we would find stronger differences between high- and low-ranking cohabiting dogs due to the subjects’ everyday experience and different relationships with the demonstrators.

**Results:**

We found that dominant dogs learn more effectively from the owner than from their subordinate dog companion. Subordinate dogs increased their success rate only when their dominant counterpart demonstrated the task, but did not improve when the owner was the demonstrator. Dogs with higher agonistic rank could improve their detour speed more often than the lower-ranked individuals in the Owner demonstration group, but we found no effect of the subranks in the Dog demonstration group.

**Conclusions:**

These results warrant the intricate effect of within-group hierarchy of dogs even in non-competitive contexts. The strong difference between the subordinate and dominant dogs’ learning performance in the Owner-demonstration group aligns with the “owner as the main resource for dogs” hypothesis.

**Supplementary Information:**

The online version contains supplementary material available at 10.1186/s12915-025-02232-9.

## Background

The social nature of dogs is a basic species-specific feature, including both their relationship with their conspecifics and, importantly, with humans [1], and it is often emphasized in comparative studies between dogs and cats (for a review see [2]).Compared to the solitary wildcat, the hypothetical ancestor of dogs was the Paleozoic wolf, which had well-developed group-living habits [3]. Given the extensive knowledge about wolves’ complex social behavior [4], one might initially assume that dogs retain a similarly rich social behavioral repertoire [5]. However, empirical evidence suggests otherwise.

In reality, compared to wolves, the intraspecific social behavior of dogs shows only moderate complexity and a more ad hoc nature even in the case of the most relevant ecotype of the species, the village (or pariah) dogs [6]. Although often living in more-or-less stable connected groups, ownerless free-roaming dogs rarely show cooperative activities such as alloparental care [7], territorial defense [8, 9] or obtaining food [10]. Based on comparative studies with similarly raised and kept, group-living, socialized dogs and wolves, it was found that while the tame wolves showed similar willingness to cooperate with their conspecifics and humans, dogs preferred to cooperate mostly with humans and not with dogs [11]. Range and Virányi [12] argued that due to domestication, the early and fundamental change in the feeding ecology of the ancestral dog population resulted in selective pressure for increased interactivity and cooperativity with humans; meanwhile, within-species competition became stronger in dogs. Among free-roaming dogs (e.g., [8, 13]), an intricate system of dominance relationships was described, where the individual dog’s rank had various components. The most direct, linear hierarchy resulted from the agonistic dominance component [13]. An additional, affiliative–submissive component was connected to “formal” dominance status, which showed a more context-specific and less linear structure than the agonistic dominance component [13], but together with the high-ranking individual’s age, could also culminate in the higher “leadership” component [14]. Beyond the free-ranging dogs and other permanently or temporarily aggregated dog groups [15], intraspecific competitive and cooperative behaviors were rarely investigated among dogs kept under close human supervision (i.e., “owned” dogs) (see e.g., [16, 17]).

Recently we introduced a new theoretical perspective about the role of the owner/caretaker as the main—social—resource for companion and working dogs to compete over [18–20]. We argued that while the classical resources involved in competitive interactions (e.g., food, shelter, mating) are usually under strong and mostly egalitarian control by the dog owner, dogs would keep competing to monopolize the attention, affection, and interactions of their owner (as seen in the case of jealous behavior: [17]). Whenever animals live in temporarily stable groups, repeated competitive interactions may result in the formation of hierarchies [21]; therefore, we proposed that the within-group hierarchies among cohabiting companion dogs may also mirror their successful access to the owner [18]. In a recent study, we showed that the summary outcome of those everyday competitive interactions between cohabiting dogs, that the owners can readily notice, was in close association with an experimentally induced competitive scenario (toy possession), and other rank-related behaviors such as greeting between conspecifics upon reunion after a short isolation [20]. Importantly, the study from Vékony et al. [20] found that the separate components of dogs’ rank score (the so-called “subscores”), had a better explanatory effect regarding the competitive behaviors of the subjects. In particular, “Agonistic” and “Formal” dominance subscores aligned well with the differences between dogs’ resource holding and intraspecific greeting behaviors. However, it was still largely unknown (see rank-related “leading” behavior during walks, [22]) whether the rank position of dogs would affect such behaviors that are not directly associated with hierarchy; however, due to their social nature, they may refer to the social dynamics within the cohabiting dog-groups.

Social learning is considered as one of the main behavioral synchronizing mechanisms between dogs and humans [1]. There is ample evidence that dogs in general show an excellent capacity for copying human actions in various problem-solving tasks [23], including the “over-imitation” of otherwise unnecessary steps within a behavioral sequence seen from a human demonstrator [24], and showing preference for a socially reinforced strategy over a more direct solution that would require trial-and-error learning [25]. Interestingly, until now, relatively few factors have been found that would affect the performance of dogs in social learning tasks (e.g., lack of breed, and age-effect in a detour task: [26]), in other words, their capacity for learning from a con- or heterospecific demonstrator can be considered as being widespread, and more or less uniform. Even the familiarity of the demonstrator with the observers was found to have an ambiguous effect in social learning tasks, with studies that did [27] and, contrarily, did not find it [28] as having a strong influence on the observer dogs’ performance. So far, only a few factors have been described as relevant influencers of the dogs’ social learning capacity. Among them was the attention-eliciting communication from the human demonstrator [29, 30], and the selective past of particular dog breeds: “cooperative” working dogs learned more effectively from a human demonstrator than “independent” working dogs did [31]. Importantly, another factor with a strong effect was the hierarchy rank of dogs: Pongrácz et al. [32, 33] found that dominant dogs did not learn from a dog demonstrator; however, they were quite capable of learning from a human demonstrator. On the other hand, subordinate dogs effectively learned from both kinds of demonstrations. However, in that original experiment, dominant and subordinate dogs from different households were tested, and the demonstrator dog and human were both previously unknown individuals for the test subjects. This made it questionable whether the results in those earlier studies could be related to the within-group rank relationships of dogs. Furthermore, although modern companion dogs routinely meet with unknown humans and dogs (e.g., [34]), the hierarchy-based evaluation of dog behavior among unfamiliar individuals can raise some concerns with regard to biological relevance.

In our present research, we opted for the robust detour paradigm coupled with social learning, but at this time the observers and demonstrators were cohabiting dogs (conspecific learning scenario) and their owners (heterospecific learning scenario). First, we assessed the rank order of cohabiting dogs (using the validated Dog Rank Assessment questionnaire (DRA-Q) described by Vékony et al. [20]); then, we tested both for their individual problem-solving ability (detour without demonstration) and in a social learning context. In the latter case, dogs from the same household served as conspecific demonstrators for each other, and the dogs’ owner functioned as the human demonstrator.

We hypothesized that similar to earlier investigations [32, 33], dogs’ rank position will be in association with their efficiency in the social learning task. However, at this time we could formulate predictions based on the fact that the participants of the social learning task were familiar with each other. We predicted that dominant dogs will not pay attention to their subordinate partner, as was found earlier in the case of other species as well (chickens: [35]; vervet monkeys: [36]; also see opposite results with chimpanzees: [37]). Based on our hypothesis of “the owner as the main limited resource for the dog,” we predicted that dominant and subordinate dogs will differ in their motivation of paying attention and relying on their owner’s demonstrating action because the dominant dogs already secured their ability to access and closely follow the owner in everyday situations. On the other hand, we predicted that subordinate dogs will learn effectively from their dominant dog partner, as was suggested by other species examples (e.g., in deer mice: [38]).

## Results

Only 35% of the dogs (29 out of 84) tested could solve the detour within 1 min in the first trial, and 24% (*N* = 20) of the dogs never made a successful detour: 27.6% of the dogs in the Control group (*N* = 8), 25% in the Dog demonstration group (*N* = 6) and 19.35% in the Owner demonstration group (*N* = 6).

### Detour success

**Fig. 1:**
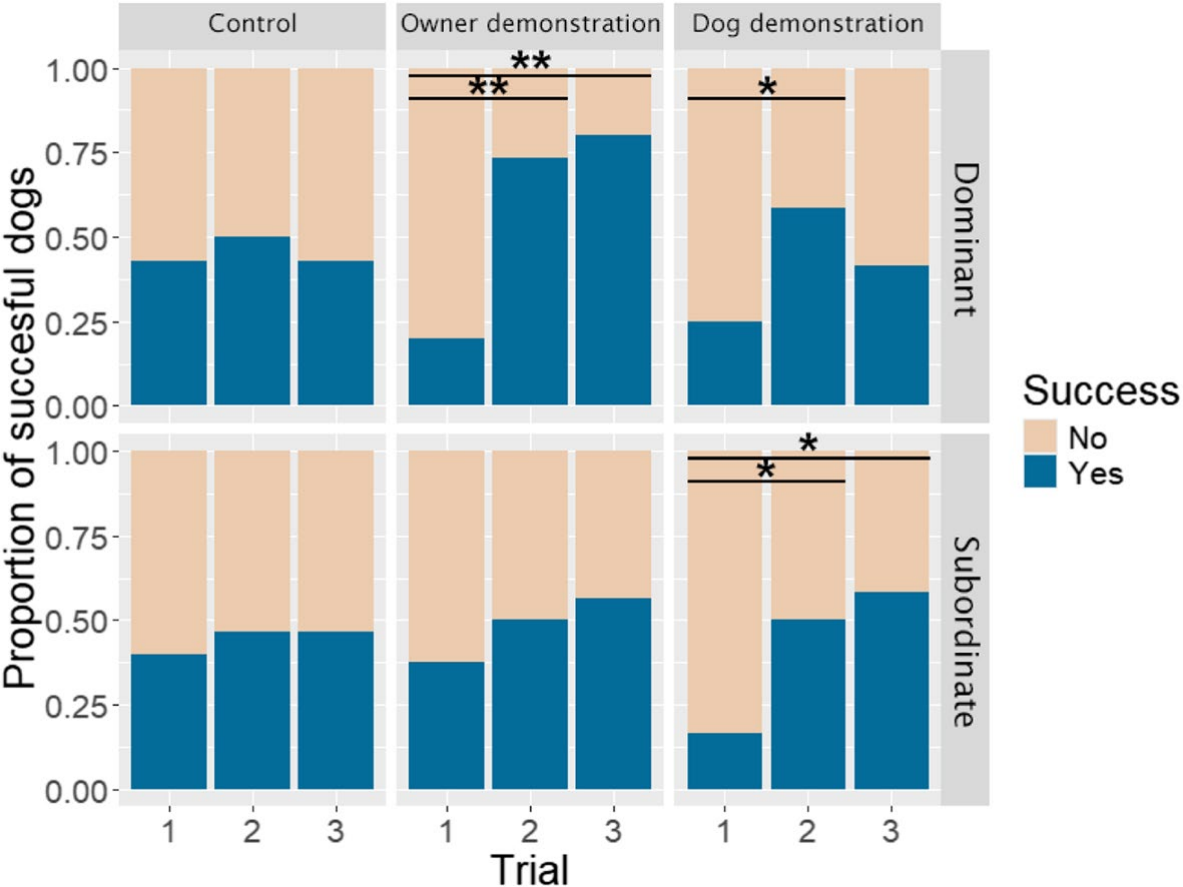
Success rates of the dominant and subordinate dogs in each group. * *p* < 0.05, ** *p* < 0.001

Cochran-*Q* test showed that dogs’ success improved across the trials only in the demonstration groups (Table 1): dogs performed better in the second and third trials than in the first one after the task was demonstrated by either the owner (Trial_1–2_: *Z* = − 2.67, *p* = 0.008; Trial_1–3_: *Z* = − 2.83, *p* = 0.005) or the cohabiting conspecific (Trial_1–2_: *Z* = − 2.83, *p* = 0.005; Trial_1–3_: *Z* = − 2.65, *p* = 0.008). No significant improvement was observed in the Control group (*p*_1–2_ = 0.317; *p*_1–3_ = 0.655). When analyzing subordinate and dominant dogs separately, we found that the success rate of subordinate dogs only improved across the trials in the Dog demonstration group (Trial_1–2_: *Z* = − 2, *p* = 0.046; Trial_1–3_: *Z* = − 2.24, *p* = 0.025), while dominant dogs more often succeeded after the demonstration in both demonstration groups (Owner: Trial_1–2_: *Z* = − 2.83, *p* = 0.005; Trial_1–3_: *Z* = − 2.71, *p* = 0.007; Dog: Trial_1–2_: *Z* = − 2, *p* = 0.046), although in the dog demonstration group there was no significant difference between the first and third trials (*p*_trial1–3_ = 0.157, Fig. 1).

**Table 1:** Results of the Cochran-*Q* tests for success rate in the three trials in each demonstration group. Statistically significant results are in bold

		Control	Owner demonstration	Dog demonstration
All dogs		*Q* = 1 *p* = 0.607	***Q* = 13.05** ***p* = 0.002**	***Q* = 12.67** ***p* = 0.002**
Rank	Dominant	*Q* = 0.4 *p* = 0.809	***Q* = 13.27** ***p* = 0.001**	***Q* = 6** ***p* = 0.050**
	Subordinate	*Q* = 2 *p* = 0.368	*Q* = 1.75 *p* = 0.417	***Q* = 8.4** ***p* = 0.015**
Formal rank	Dominant	*Q* = 1.5 *p* = 0.472	***Q* = 6.5** ***p* = 0.039**	*Q* = 4.5 *p* = 0.105
	Subordinate	*Q* = 0 *p* = 1	*Q* = 4.2 *p* = 0.122	***Q* = 6** ***p* = 0.050**
Agonistic rank	Dominant	*Q* = 0.5 *p* = 0.779	***Q* = 16.8** ***p* < 0.001**	***Q* = 7.6** ***p* = 0.022**
	Subordinate	*Q* = 0 *p* = 1	*Q* = 1.75 *p* = 0.417	***Q* = 6.5** ***p* = 0.039**
Leadership rank	Dominant	*Q* = 3 *p* = 0.223	***Q* = 11.4** ***p* = 0.003**	***Q* = 6** ***p* = 0.050**
	Subordinate	*Q* = 2 *p* = 0.368	*Q* = 3.5 *p* = 0.174	*Q* = 4.67 *p* = 0.097

**Fig. 2:**
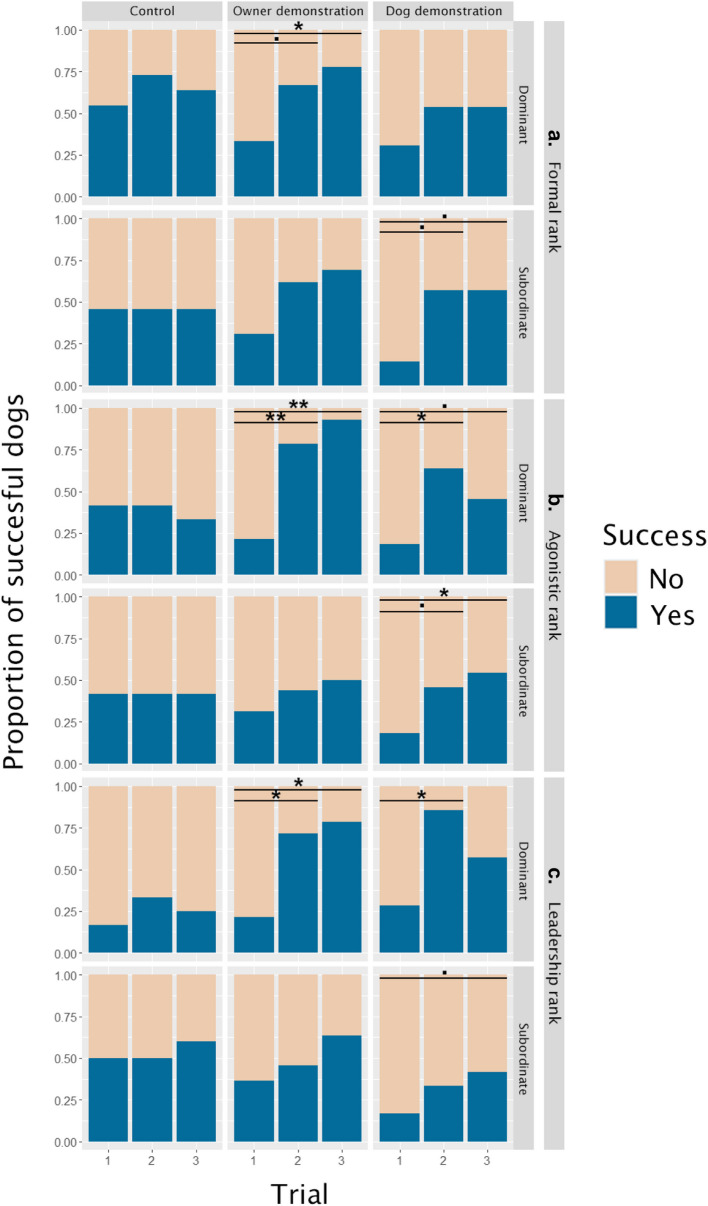
Success rates in each demonstration group based on subranks: (**a**) formal rank, (**b**) agonistic rank, and (**c**) leadership rank. *p* < 0.1, * *p* < 0.05, ** *p* < 0.001

When we used the subranks, the Cochran-*Q* tests revealed a similar pattern to the one seen in the case of holistic rank (Table 1): Formally dominant dogs’ success rate only improved after owner demonstration (Trial_1–2_: *Z* = − 1.73, *p* = 0.083; Trial_1–3_: *Z* = − 2, *p* = 0.046), while subordinates only showed a trend-like improvement in the dog demonstration group (Trial_1–2_: *Z* = − 1.73, *p* = 0.083; Trial_1–3_: *Z* = − 1.73, *p* = 0.083, Fig. 2a).

Agonistically dominant dogs were more successful after the demonstration in both demonstration groups (Owner: Trial_1–2_: *Z* = − 2.83, *p* = 0.005; Trial_1–3_: *Z* = − 3.16, *p* = 0.002; Dog: Trial_1–2_: *Z* = − 2.24, *p* = 0.025; Trial_1–3_: *Z* = − 1.73, *p* = 0.083), while subordinates only succeeded more after the dog demonstration (Trial_1–2_: *Z* = − 1.73, *p* = 0.083; Trial_1–3_: *Z* = − 2, *p* = 0.046, Fig. 2b).

Dominant leaders succeeded more after both demonstrations (Owner: Trial_1–2_: *Z* = − 2.33, *p* = 0.020; Trial_1–3_: *Z* = − 2.53, *p* = 0.011; Dog: Trial_1–2_: *Z* = − 2, *p* = 0.046), although trial 3 did not differ from trial 1 in the dog demonstration group (*p*_trial1–3_ = 0.157), while the followers’ success rates marginally improved after the other dog demonstrated the task twice (Trial_1–3_: *Z* = − 1.73, *p* = 0.083, Fig. 2c).

### Latency of reaching the reward

#### Between-group differences

**Fig. 3:**
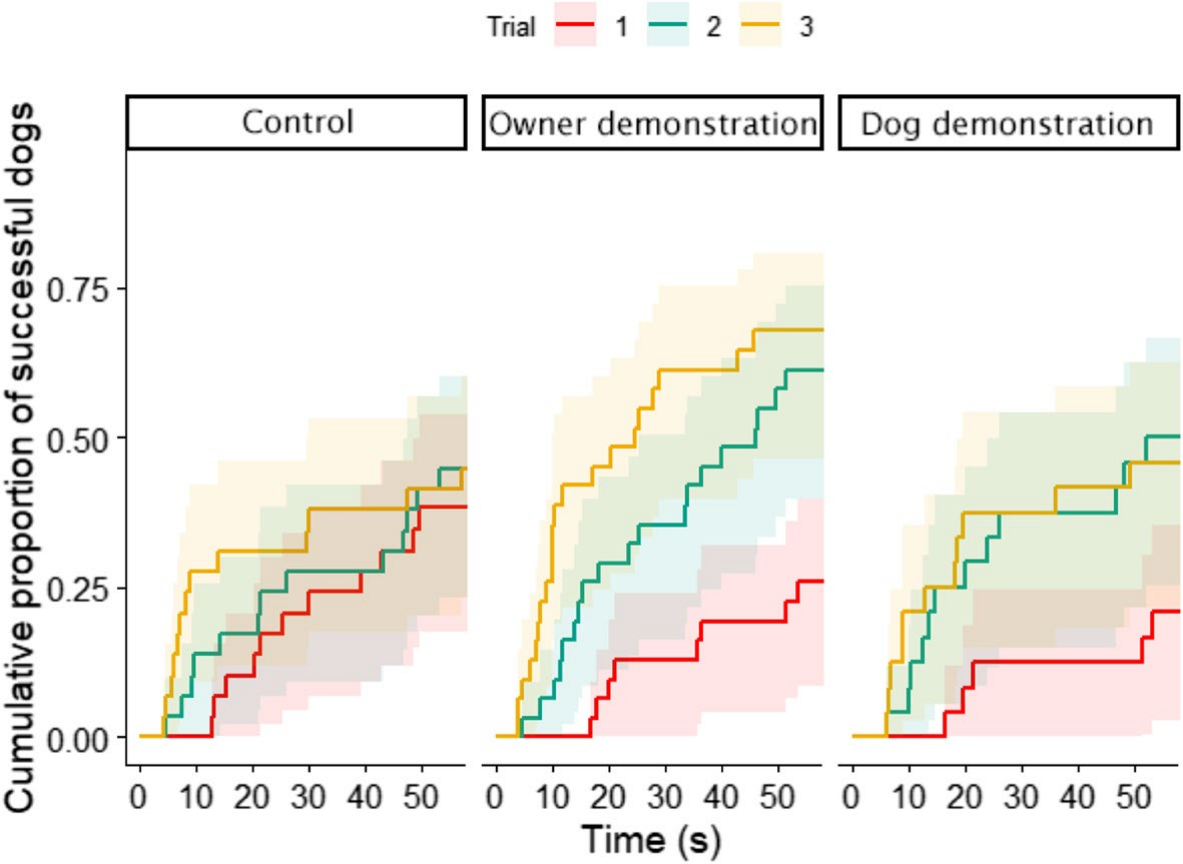
Detour latencies across the trials in each demonstration group. Dogs improved in Trial 2 in both demonstration groups and further improved in Trial 3 of the owner demonstration group

Based on the results of all dogs in our sample, we first analyzed if there was a difference in the improvement of dogs’ detour latencies between groups. We found an interaction between trial number and experimental group. Dogs in the Control group only improved in the third trial (Trial_1–3_: β ± SE = − 1.530 ± 0.50, *z* = − 3.05, *p* = 0.007), while in the Dog demonstration group, dogs made a detour significantly faster in both trials after demonstration than in the first trial (Trial_1–2_: β ± SE = − 2.348 ± 0.57, *z* = − 4.15, *p* < 0.001; Trial_1–3_: β ± SE = − 2.412 ± 0.59, *z* = − 4.07, *p* < 0.001). Their latencies did not differ between the second and third trials (*p* = 0.99). In the Owner demonstration group, there was a significant difference between each trials’ detour latencies, with steady improvement from trial 1 to trial 3 (Trial_1–2_: β ± SE = − 1.923 ± 0.45, *z* = − 4.25, *p* = 0.0001; Trial_1–3_: β ± SE = − 2.977 ± 0.48, *z* = − 6.20, *p* < 0.001; Trial_2–3_: β ± SE = − 1.054 ± 0.37, *z* = − 2.86, *p* = 0.012, Fig. 3).

#### Effects of rank within groups

##### Control group

When using the holistic rank and rank score in our analysis, in the Control group we only found a significant interaction between trials and the age of the dogs: older dogs in general were slower in solving the detour, but according to the post hoc test they improved more after the first trial (Trial_1–2_: β ± SE = − 0.621 ± 0.28, *z* = − 2.25, *p* = 0.062; Trial_1–3_: β ± SE = − 0.823 ± 0.30, *z* = − 2.76, *p* = 0.016).

When investigating the subranks and subscores separately, we found a similar interaction between trial and age (Trial_1–2_: β ± SE = − 0.591 ± 0.27, *z* = − 2.20, *p* = 0.071; Trial_1–3_: β ± SE = − 0.784 ± 0.28, *z* = − 2.76, *p* = 0.016). The best model also contained each of the three subranks, although their effects pointed in opposite directions: Formally dominant dogs made the detour faster than both the subordinate (β ± SE = 3.670 ± 1.07, *z* = 3.42, *p* = 0.002) and flexible ranked dogs (β ± SE = 5.370 ± 1.86, *z* = 2.89, *p* = 0.011). Agonistically dominant dogs, on the other hand, were slower than the subordinates (β ± SE = − 4.885 ± 1.32, *z* = − 3.69, *p* < 0.001) or flexible ranked dogs (β ± SE = − 4.430 ± 1.41, *z* = − 3.15, *p* = 0.005), and a similar effect was found in the case of Leadership rank (Dom-Sub: β ± SE = − 3.570 ± 1.29, *z* = − 2.76, *p* = 0.016; Dom-Flex: β ± SE = − 2.990 ± 1.22, *z* = − 2.45, *p* = 0.038). This model had a better fit than the one without subranks (∆AICc = 10.44, *p* < 0.001).

##### Owner demonstration

In the case of Owner demonstration, the holistic rank score did not have a significant effect; only the Trials showed a significant association with the detour latencies. Dogs improved not only between the first and second (β ± SE = − 1.820 ± 0.47, *z* = − 3.85, *p* < 0.001) and the first and third (β ± SE = − 2.920 ± 0.51, *z* = − 5.72, *p* < 0.001) but also between the second and third trials (β ± SE = − 1.090 ± 0.37, *z* = − 2.94, *p* = 0.009).

**Fig. 4:**
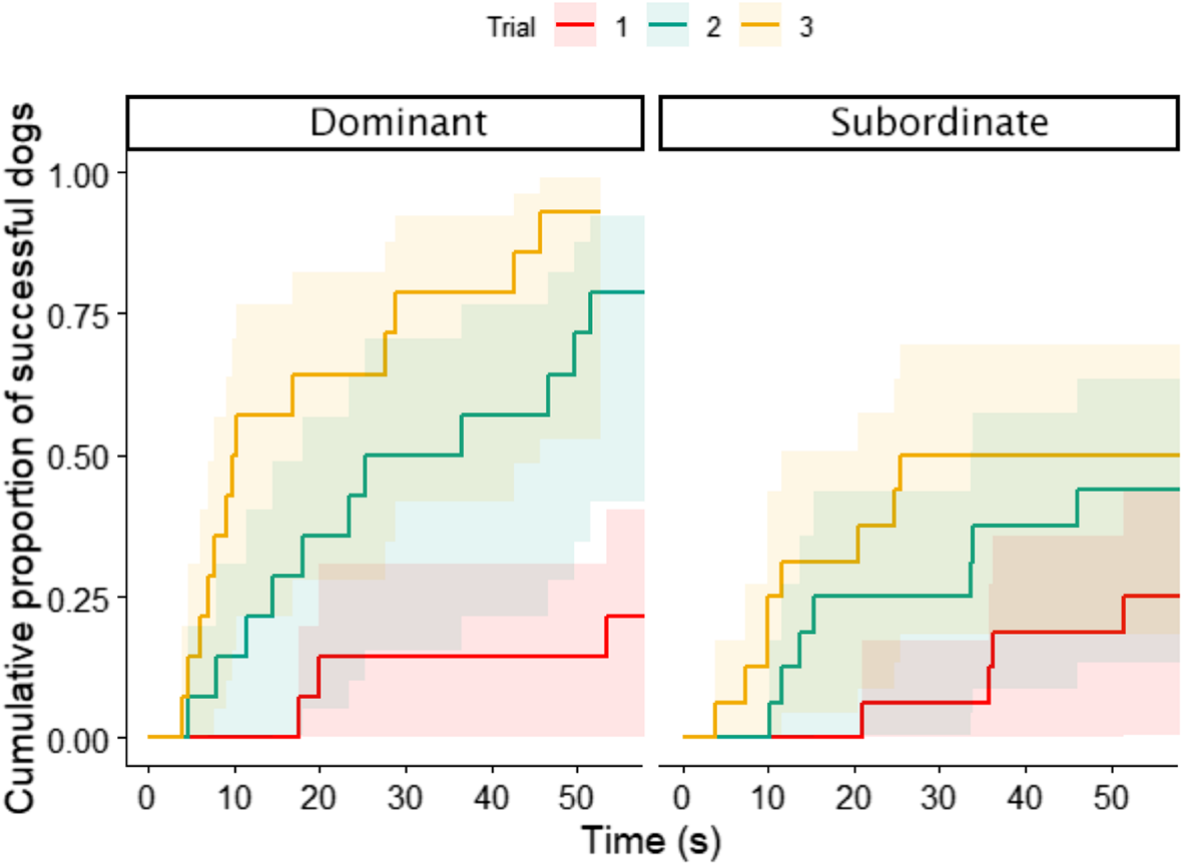
Improvement of latency of the detour by trial and by agonistic rank in the owner demonstration group. Dominant dogs’ latencies improved right after the first demonstration, and they became faster with each trial, while subordinate dogs only improved after the second demonstration

Trials had a similar effect in the models with subranks as an independent factor. Agonistic rank was in an interaction with trial: while agonistically dominant dogs improved in all trials (Trial_1–2_: β ± SE = − 2.573 ± 0.74, *z* = − 3.49, *p* = 0.001; Trial_1–3_: β ± SE = − 4.023 ± 0.78, *z* = − 5.18, *p* < 0.001; Trial_2–3_: β ± SE = − 1.450 ± 0.49, *z* = − 2.97, *p* = 0.009), subordinate dogs’ improvement was only significant from the first to the third trial (β ± SE = − 1.917 ± 0.65, *z* = − 2.97, *p* = 0.008). In their case, no significant difference was found between other trials (*p*_1–2_ = 0.13; *p*_2–3_ = 0.41, Fig. 4). This model had a better fit (∆AICc = 2.48, *p* = 0.027).

##### Dog demonstration

In the Dog demonstration group, only trial had a significant effect in all final models. Dogs solved the detour faster after the demonstration in both the second (β ± SE = − 2.875 ± 0.68, *z* = − 4.28, *p* < 0.001) and third trial (β ± SE = − 2.950 ± 0.71, *z* = − 4.19, *p* < 0.001) compared to the first.

## Discussion

In this study, we used the well-established detour paradigm to investigate how rank relationships between cohabiting family dogs might affect their social learning ability from each other and the owner. While the possible effect of rank on social learning was previously investigated by Pongrácz and colleagues [32, 33], the demonstrators in those studies were either an unfamiliar experimenter or an unfamiliar dog, making the situation harder to interpret for everyday life situations. The other difference to previous social learning detour studies was that they usually only used the latency of the detour as a measure of improvement from trial to trial, but we decided to also use a much simpler measure: the binary variable of dogs’ success in reaching the treat.

Pongrácz and colleagues [28] mentioned in their first detour study that this task is difficult for dogs, and our results highlight this notion even more with the high proportion of unsuccessful dogs in every condition.

The success rates largely aligned with our predictions. Subordinate dogs obtained the reward with increasing success rates across the trials only when the other (dominant) dog was the demonstrator; meanwhile, their success rate did not change when their owner showed how to detour the fence. The dominant dogs, in general, performed better than the subordinate ones; however, in their case, the owner especially served as a salient demonstrator, and the dominant dogs’ improvement was only modest when they observed the demonstration of their lower-ranking companion. Regarding the success rates, the holistic rank and the three subscores had very similar associations with the dogs’ performance. The effect of the three subscores always pointed in the same direction, with only the “formally dominant” dogs showing no significant improvement in the case of the dog demonstrator. When we compare these results to the similar earlier study [32], the new findings mainly confirmed the earlier results. Although, in the first study, the researchers only analyzed the detour latencies, they also found that dominant dogs learned more successfully from the human demonstrator, while the subordinate animals mainly excelled after observing the demonstrator dog’s action.

Compared to the earlier study, we also found some differences. In the present experiment, higher-ranking dogs were not completely unable to learn from the other dog: although to a lesser extent than after observing their owner, they still improved after the dog demonstration. Even more strikingly, now we found that lower-ranking dogs were not improving at all in their success rates after observing the demonstration by their owner, while in previous studies, they successfully learned from a stranger [32]. As in our current test, detour latencies did not (or only minimally) associate with the rank of the dogs; the above-mentioned differences between the findings of Pongrácz et al. [32] and this study cannot be attributed solely to the different analyzed parameters, but rather to the familiarity of demonstrators.

The result that lower-ranking dogs did not learn from the owner at all, meanwhile the higher-ranking dog from the same household did, can be considered as the clearest indication of the"owner as a resource’ hypothesis thus far [18–20]. This idea strongly differs from the theory that places humans (i.e., the owner) within the hierarchy of dogs at home (e.g., reviewed by [39]). In our opinion, and according to others (e.g., [40, 41]), in the case of companion dogs, it is biologically not meaningful to model the dog–human relationship in the framework of competitive hierarchy establishment. Contrary to this, cohabiting dogs compete for exclusive access to the owner’s attention and care (see also: jealousy-like behaviors, [17]). From this aspect, in established rank systems, higher-ranking dogs may get used to the primary access and interactions with the owner, while lower-ranking individuals could learn to be more independent of the owner’s influence. In our test, if the lower-ranking dogs showed lower levels of human dependence, this can cause weaker performance in the social learning task, as was found in the case of those dog breeds that have been selected for independent working tasks [29, 31]. Using cohabitant dogs as subjects and the owner as a demonstrator, instead of randomly chosen dominant and subordinate dogs and an unfamiliar experimenter, as in the case of Pongrácz et al. [32], could result in the stronger contrast between the efficiency of learning from the demonstrators in our current experiment. While subordinate dogs may turn towards an unfamiliar person with interest and willingness to follow, the demonstration performed by their owners could evoke only minimal interest because of the effect of learned disadvantage against their dominant dog companion.

The most striking result of the earlier study run by Pongrácz et al. [32] was that dominant dogs did not learn from the (unfamiliar) demonstrator dog at all, how to effectively detour the fence. Now we found that higher-ranking dogs excelled in learning from the owner, and they also showed modest improvement by observing the familiar, lower-ranking dog. This result highlights the potential relevance of familiarity among demonstrators and observers in the case of intraspecific social learning in dogs. Demonstrator-observer familiarity is a core requirement in most social learning studies on various species (e.g., budgerigars, [42]; guppies, [43]; rats, [44]). Dogs can be regarded as an exception, as they were found to be flexible in how readily they accept unfamiliar humans and dogs as demonstrators [24, 28, 32, 45]. However, as our present study has shown, cohabitant dogs can learn to pay attention to each other at home, and this may cause even the higher-ranking individuals to learn from their familiar canine group members.

In another study, dogs’ rank was found to be associated with some of their personality traits [19]. It would be interesting to know whether dogs’ personality could explain the differences between high- and low-ranking dogs’ performance in the detour task. According to Vékony and colleagues’ findings [19], high-ranking dogs could be characterized by features such as persistence, focus, efficiency (belonging to Conscientiousness); curiosity (belonging to Openness); and activity, assertiveness (belonging to Extroversion). Although these characteristics could result in better problem-solving performance (e.g., [46]), we found a lack of rank effect in the control group. This suggests that the rank-related differences found in the other test groups could be caused by such confounders, as higher drive to explore, or generally better problem-solving capacity.

The results based on latencies partially confirmed the earlier findings. Detour latency in the owner demonstration group showed a similar association with the Agonistic score as did the previous study by Pongrácz and colleagues [32]. While higher-scoring dogs improved their detour speed right after the first demonstration, lower-ranking dogs only improved after two demonstrations. Interestingly, the rank assessment questionnaire of the earlier study only consisted of four questions, and the scoring of the questions put more weight on agonistic behaviors (i.e., the dog winning fights could not be categorized as subordinate), which might explain why the Agonistic subscale of the currently used DRA-Q shows more similarity with the previous results than Rank as a whole.

However, in the dog-demonstration group, we did not find any effect of the rank; instead, all dogs became faster through the repeated trials, showing a general (and expected) effect of social learning from the demonstrator (similar to the results of [30, 47]). Compared to the results of the present study, earlier it was found that the dominant dogs were not able to improve their detour latencies at all when another dog demonstrated the task, while the subordinate dogs performed the detour very fast right from the beginning [32]. The familiarity between the demonstrator and observer dog in this present experiment could result in somewhat higher attention towards the demonstration in the higher-ranking dogs, which, coupled with the relatively low sample size, could result in a disappearing rank-effect.

While in other studies the subranks had divergent associations and their relevance was strongly context-dependent [20, 48], here they point to the same direction in the case of success rate. This suggests that social learning might be one of the few contexts where all aspects of rank have similar relevance. Additionally, the non-competitive aspect of the detour task and the demonstrators’ action could also be the reason why the various components of dominance did not affect differently the dogs’ behavior.

So far, no previous study has shown that any intrinsic factor would affect dogs’ performance in the detour task without demonstration, apart from the spatial arrangement of the obstacle (“outward” detour vs. “inward”: [28]; straight fence vs. V-shaped one: [49]). There are indications in the literature that dogs’ sex could be a confounder in various problem-solving tasks that include training and interaction with humans (e.g., female dogs responded better to gestural commands than male dogs did: [50]; male dogs received higher “boldness” scores and initially performed better in working dog trials than females did: [51]). However, we did not find any association among our parameters with the dogs’ sex, which was possibly because our samples were well-balanced between the two sexes. Similar to previous studies, the holistic rank score did not show any association with detour latency in the control group. Subranks, however, seem to affect individual problem-solving in different ways. Formally dominant dogs were faster to solve the detour on their own, while agonistically dominant dogs and leaders seem to be slower. This result provides intriguing insight into the potential association between particular subrank components and spatial problem solving. The dominance-component “leadership” involves higher activity levels and alertness, and together with “agonistic” predicts a more assertive dog—however, these characteristics are seemingly of little use to effectively solve the detour task. This result fairly resembles the somewhat surprising findings of Dobos and Pongrácz [29, 31] which showed that dog breeds that were originally selected for independent work tasks were not more successful on their own in the detour task than the cooperative, more human-dependent breeds were. However, dogs with higher “formal dominance” scores are mainly characterized by receiving active submission from their peers. The relatively higher performance of these dogs in the control condition can possibly be the result of their generally higher experience in problem solving. Formal dominance can be regarded as the most “established” rank status because it is based on the other group members’ respect [13]. Such positions in the hierarchy can be the result of not only assertiveness and strength but also experience and age.

## Limitations to the study

The main limitations of this study affect the dog demonstration group. This was our smallest group due to difficulties in training demonstrator dogs. The success rate was generally low in our sample (24% of dogs did not have any successful trials), leaving fewer possible demonstrator dogs in the subject pool. Some dogs that had successful trials lost interest in the task, so they could not be used as demonstrators for their cohabiting conspecifics. In previous studies with dog demonstrators [32, 52], the unfamiliar demonstrator dogs were specifically chosen to be easily trained to solve the detour task and stay motivated after multiple demonstrations, but in our case, the amount of training was limited and the motivation of the cohabiting dogs was non-modifiable.

This group also differed from the control and owner demonstration groups because not only the focal dog, but the (familiar) demonstrator also had a Rank score, and these scores were mutually interdependent with each other. Our understanding of Rank scores is that the differences between scores of cohabiting dogs describe the steepness of the hierarchy between them, but using the score differences instead of the Rank scores would make comparisons between the experimental groups impossible, as there are no such measures in the control and owner demonstration group.

Another limitation stems from the questionnaire itself. While previous studies have shown that formal submissive displays are the most unambiguous indicators of rank [13, 53], in our case, only one item of the DRA-Q refers to the formal rank. This, especially with the option of the “not applicable” response, made it impossible to use Formal score in the latency analyses.

## Conclusions

Our findings suggest that social ranks in cohabiting dogs may have relevance in non-competitive scenarios such as learning from the owner and their cohabiting conspecifics. Contrary to previous findings, dominant dogs effectively learned from their cohabiting dog’s demonstration, although their success rate improved less when subordinate dogs observed the high-ranking dog demonstrator. This suggests that the familiarity of the demonstrator is an important factor in social learning from conspecifics even in the case of very socially flexible dogs. This effect was previously tested and has not been found in the case of human demonstrators.

The marked difference between the high- and low-ranking dogs’ improvement in their success rates after observing the owner demonstration suggests a qualitative, rank-related difference in motivation and relationship with the owner in the cohabiting dogs. This finding supports our notion that relationship with the owner—in the absence of other limited resources—might be the driving force behind forming hierarchies among cohabiting companion canines.

## Methods

### Subjects

Our subjects were companion dogs from multi-dog households (i.e., the owner had at least two dogs at home). The owners always participated with two of their dogs in our tests. The owners first completed the DRA-Q questionnaire that we used to determine the holistic Rank scores and Formal, Agonistic, and Leadership subscores of their dogs [20].

We tested *N*_tested_ = 95 dogs, but had to exclude some of them from the video coding (we excluded one subject due to the owner not following the protocol, two because of technical issues with the cameras, and five dogs did not even try the detour). We coded the videos of *N*_coded_ = 87 dogs (40 males, 47 females, *M*_age_ ± SD = 5.75 ± 3.43), but excluded three dogs from the final analyses as they solved the detour in the first trial under 10 s, leaving little room for improvement in the subsequent trials. We included *N*_analyzed_ = 84 dogs in the statistical analyses (Tables 2 and 3).

**Table 2:** Number of dogs in each group by their relative rank. The data from these dogs were included in the statistical analysis

	Owner demonstration	Dog demonstration	Control	
Dominant dog	15	12	14	41
Subordinate dog	16	12	15	43
	31	24	29	**84**

**Table 3:** Number of dogs in each group by their sex and relative rank. The data from these dogs were included in the statistical analysis. *DomMale*, dominant male; *SubMale*, subordinate male; *DomFemale*, dominant female; *SubFemale*, subordinate female

	DomMale	SubMale	DomFemale	SubFemale	
Owner	7	8	8	8	31
Dog	7	5	5	7	24
Control	7	5	7	10	29
	21	18	20	25	**84**

### Experimental setup

**Fig. 5:**
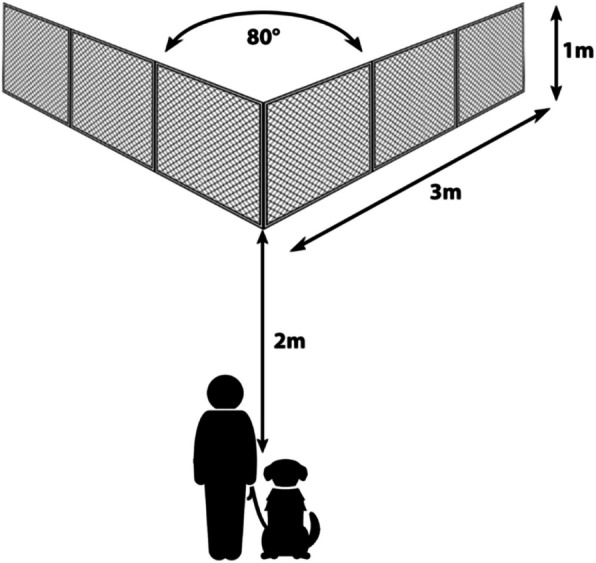
Experimental setup of the detour test (taken from Pongrácz et al. 2021)

The tests were conducted in an approximately 10 × 10 m, enclosed, outdoor area at a dog school in Budapest, Hungary. We always ran the tests when there was no other *training activity* in the dog school and no other dogs could approach the test area. The transparent V-shaped fence (made of wire mesh stretched over a light steel frame, 3 m each side, 1 m high) was set up so its sides formed an approximately 80° angle and there were at least 3 m between the ends of the wings to the closest border of the enclosure (Fig. 5).

The starting line was marked with two plastic cones 2 m from the corner of the fence, and a white plastic plate was placed at the inner corner of the fence, so any reward used during the tests would be more visible for the dogs.

The tests were recorded from both sides, with a wide-angle action camera (GoPro Hero4 or Lamax 9.1 W) and a regular camera (Canon Powershot SX432 or Sony HDR-CX450).

### Procedure

The test protocol was based on previous studies of Pongrácz and colleagues [18, 28, 32]. The dogs were tested in three experimental groups: Control group, Owner demonstration group, and Dog demonstration group. Within these groups, we aimed to have a similar number of dominant and subordinate dogs, based on their Rank scores (compared to the other participating dog from the same household). We tested the two dogs of each owner on two separate occasions.

#### Control group

The test consisted of three identical trials: the experimenter (E) kept the dog on leash at the starting point while the owner (O) called the dog’s attention and showed a treat or a toy (based on the owner’s choice, whichever reward motivated the dog the most), then walked to the corner of the V-shaped fence while keeping the dog’s attention on the reward, and then placed the reward on the plate in the inner corner of the fence from above. Then the O walked back to the dog, took the leash from the E and released the dog with a release cue (e.g., “Go get it!”). The O was asked to use verbal encouragement throughout the trials, but they were not allowed to use any verbal or gestural commands related to directions (e.g., “Forward,” “Turn,” “Go around,” “Left,” “Right”). Each dog had 60 s from being released to obtain the reward. After obtaining the reward, or if the 60 s elapsed without a successful detour, the O called the dog back and put them on leash.

#### Owner demonstration group

The first trial was identical to that of the Control group, but in the second and third trials, the O, instead of dropping the reward to the plate from above, walked around one side of the fence while keeping the dog’s attention using ostensive verbal cues (e.g., “Look, here it is”), placed the reward on the plate and walked out along the other wing of the fence (i.e., walking a “heart-shaped” path during demonstration). The direction of the detour was chosen randomly. After the demonstration, the trials were run identically, as it was done before (i.e., releasing the dog, measuring the time).

#### Dog demonstration group

The demonstrator in this group was always the cohabitant dog of the tested dog (TD). The cohabitant dog previously participated in the detour test, either in the Control group or Owner demonstration group. Before testing, the TD was taken for a walk while the demonstrator dog (DD) was further trained to reliably detour around the fence. The training lasted until the demonstrator dog detoured around the fence without any stopping or hesitation in three consecutive trials. The maximum length of the training was 15 min. If the demonstrator dog could not be trained, the TD was reassigned to the Control or Owner demonstration group.

The first trial for the Dog demonstration group was identical to the one described in the case of the Control and Owner demonstration groups. In the second and third trials, both dogs stood on the starting line (they were both on leash and the E held them there) while the O called their attention, showing the reward and placing it on the plate in the corner from above. After walking back, the O released the DD, while encouraging the TD to pay attention to the detour. After the DD successfully obtained the reward and was put back on leash, the owner put another reward in the corner of the fence while calling the TD’s attention, then released the TD, who again, had 60 s to obtain the reward.

### Behavioral coding

After synchronizing the videos of the two cameras, the tests were coded with BORIS (v 7.13.6 © Olivier Friard and Marco Gamba [54]). Table 4 shows the coded behaviors.

**Table 4:** These variables were coded for each trial

Variable	Description	Measure
Success	The dog could or could not reach the reward in 60 s	0/1
Latency	Time elapsed from when the owner let the dog go until the dog touched the reward (60 s in the case of unsuccessful trial)	s

### Statistical analyses

Statistical analyses were performed by using R statistical software (v4.3.0, R Development Core Team, 2021) in RStudio (2024.04.2 + 764, RStudio Team, Boston, MA, USA) with packages AICcmodavg, coxme, emmeans, glmmTMB, lme4, lmerTestm, MuMIN, performance, rstatix, survival, and survminer. We used the Cochran-*Q* test to assess whether the success rates of dominant versus subordinate dogs differed across the trials within each group. This test was chosen because it is specifically designed for analyzing categorical data and comparing the frequencies of success between multiple trials in the same subjects. By applying this test, we were able to determine if the performance of dogs, as categorized by dominance status, improved over the course of the trials in each group. We used Mixed Effects Cox Regression models to analyze the association between Latency and dogs’ rank. We also included the age of the dog as an independent variable in the initial models. We used AIC-based model selection to find the parsimonious model. We performed the model selection with the holistic Rank and Rank score, then the subscores and subranks separately, and finally, we compared the final models to find the best fit.

## Supplementary Information

Supplementary Material 1.

## Data Availability

All data generated or analyzed during this study are included in this published article [and its supplementary information file Supplement_data_raw_Hierarchy_detour.xlsx].

## Abbreviations

DRA-Q: Dog Rank Assessment Questionnaire
SE: Standard error
DomMale: Dominant male
SubMale: Subordinate male
DomFemale: Dominant female
SubFemale: Subordinate female
E: Experimenter
O: Owner
TD: Tested dog
DD: Demonstrator dog
